# Prevalence of *Strongyloides stercoralis* infection and hyperinfection syndrome among renal allograft recipients in Central Europe

**DOI:** 10.1038/s41598-018-33775-3

**Published:** 2018-10-18

**Authors:** Wolfgang Winnicki, Michael Eder, Peter Mazal, Florian J. Mayer, Gürkan Sengölge, Ludwig Wagner

**Affiliations:** 1 Department of Internal Medicine III; Division of Nephrology and Dialysis, Medical University of Vienna, Vienna, Austria; 2 Department of Clinical Pathology, Medical University of Vienna, Vienna, Austria; 3 Department of Laboratory Medicine, Medical University of Vienna, Vienna, Austria

## Abstract

*Strongyloides stercoralis* is not hyperendemic in European countries but has been increasing in prevalence due to migration and travel. The infection is characterized by a mostly asymptomatic course or nonspecific symptoms in healthy subjects. However, immunosuppression or chemotherapy have been described as leading triggers for *Strongyloides stercoralis* hyperinfection syndrome and may have a fatal course. A post hoc analysis was performed among renal transplant patients during a 5-year period. Plasma samples of two hundred kidney allograft recipients were retrospectively analyzed for *Strongyloides stercoralis* seropositivity by established ELISA testing. Positive *Strongyloides stercoralis* serology was found in 3% of allograft recipients. One patient developed a life-threatening hyperinfection syndrome. His *Strongyloides* IgG signal had been elevated for years before the outbreak of the disease. *Stronglyoides* infections in transplant recipients are an important issue that physicians also in Central Europe should be aware of, given the risk of hyperinfection syndrome and the challenges in clinical diagnosis. Our study suggests that recipient and donor screening should be recommended in kidney transplantation programs in Central Europe as S*trongyloides* infection rates increase and its prevalence may be underestimated. Further research is needed to understand why some *Strongyloides stercoralis* seropositive individuals develop hyperinfection syndrome and others do not.

## Introduction

*Strongyloides stercoralis* (*S. stercoralis*) is a parasite causing an enteric infection in animals and humans. It is hyperendemic mostly in the tropics and subtropics with a prevalence of over 70% in selected countries^1^ and is widely distributed, affecting up to 370 million humans worldwide^1,2^. Genetic analysis has shown that there are genotypic variations in *S. stercoralis* populations and global diversity. There are two lineages of *S. stercoralis*, termed type A and type B^3^. Type A causes infections in humans and dogs, while type B infects only dogs^4^. Chronically infected humans frequently do not show symptoms but can develop a disseminated disease following immunosuppressive or chemotherapeutic treatment^5,6^. The frequently asymptomatic or nonspecific nature of active S*trongyloides* infection leads to underdiagnosis and places immunosuppressed patients at increased risk of developing hyperinfection and adverse outcome^7^. It is therefore a challenge for physicians who treat patients with immunosuppressive regimens such as allograft recipients, patients with autoimmune diseases and individuals after chemotherapy^8^.

Several serological tests have been validated for clinical use recently. Four of them have a good level of specificity and sensitivity for the diagnosis of infected individuals^9^. However, low level or dormant infections might still be underestimated using these tests. Whether real-time quantitative PCR based stool testing^10–12^ provides better results, especially in patients with low number of parasites, is still unknown.

Case reports have documented hyperinfection syndrome with *S. stercoralis* in immunosuppressed kidney allograft recipients at a time when cyclosporine was the standard of care^13,14^. At present, immunosuppressive regimens mainly consist of tacrolimus together with steroids and mycophenolate in kidney transplant patients. This could predispose to *S. stercoralis* infections, since cyclosporine has a proven anthelmintic potential^15^, while tacrolimus has no antiparasitic effect against *S. stercoralis*^16^. The global distribution of the helminth has been well documented in literature and so far, Central Europe has not been described as a high endemic area^1^. However, the incidence of strongyloidiasis is increasing regionally in Europe^17^. This can be explained by an increasing number of travelers to and migration from endemic countries and an increase in immunosuppressed patients^17^. These facts should alert transplant physicians to start screening for parasitic infections even in asymptomatic patients at risk before enrollment into transplant procedures and start of immunosuppressive therapy. Furthermore, since the transmission of *S. stercoralis* by kidney transplantation has already been demonstrated, screening not only of high-risk recipients but also of donors for *Strongyloides* infections would be a valid approach to prevent outbreaks of hyperinfection syndromes and the further spread of the disease^18,19^.

Motivated by a nearly fatal course of disease of one individual who presented with *S. stercoralis* hyperinfection syndrome and the fact that routine *S. stercoralis* screening was not yet part of pretransplant screening protocols in our center, this investigation was initiated covering transplant recipients from the city of Vienna, Austria, and its surroundings. In this study we have chosen one of the tests currently available in Europe for 1) evaluating the prevalence of *S. stercoralis* infection and seropositivity among renal allograft recipients, 2) testing whether patients with migrant background are more frequently carriers of the parasite, 3) investigating the course of immunoglobulin G (IgG) signal over time post transplantation, while triple immunosuppressive therapy represents the standard of care and 4) following the antibody signal of the patient developing disseminated disease over years.

## Methods

### Study design

This retrospective case-control study was designed to evaluate the prevalence of *S. stercoralis* infection and seropositivity among renal allograft recipients under triple immunosuppressive therapy at our Division of Nephrology and Dialysis at the Medical University of Vienna, since routine *S. stercoralis* screening was not yet part of our pretransplant screening protocols. Furthermore, patients’ plasma samples were investigated over a period of one to 5 years to examine whether infection spreading occurred in the course of constant immunosuppression. All plasma samples were blinded by numbers for anonymization and had been kept on −80 °C until analysis.

### Study population

Two hundred adult renal allograft recipients (female/male = 80/120, mean age at transplantation 55.9 ± 12.3 years), transplanted in our center were enrolled on a consecutive basis. Blood samples were withdrawn four weeks after transplantation and at routine check-ups for renal function and tacrolimus level controls at our outpatient unit. Patients living in the city of Vienna, the capital city of Austria, or its close surroundings were enrolled. The primary sample collection was carried out between 01/2010 and 05/2013 and follow-ups were performed until 01/2017. Forty-five patients were of migrant background, defined as individuals born abroad who moved to the study site from the former Yugoslavia, Turkey or East Asia. The other 155 patients were Austrian born inhabitants. Demographics of the study population are given in Table 1. All patients passed a specific health checkup, which included cardiography, chest X-ray, blood tests for liver function as well as hematological testing and serological evaluations for alloantigen specific antibody presence. None of the enrolled patients had a relative eosinophil count higher than 5%. Patients older than 50 years had undergone a colonoscopy.

**Table 1:** Demographics of study population.

Characteristics	All patients n = 200	Migrant patients n = 45	Non-migrant patients n = 155	p-value
Age at transplantation, (years)	55.9 ± 12.3	52.2 ± 11.7	56.9 ± 12.3	0.022
Female recipient, n (%)	80 (40.0)	20 (44.4)	60 (38.7)	0.489
First renal allograft, n (%)	167 (83.5)	40 (88.9)	127 (81.9)	0.269
Repeated renal allograft, n (%)	33(16.5)	5 (11.1)	28 (18)	0.526
**Cause of end-stage renal disease**				**0.063**
Glomerulonephritis, n (%)	44 (22.0)	4 (8.9)	40 (25.8)	
Diabetic nephropathy, n (%)	29 (14.5)	5 (11.1)	24 (15.5)	
Hypertension, n (%)	13 (6.5)	4 (8.9)	9 (5.8)	
Cystic kidney disease, n (%)	31 (15.5)	6 (13.3)	25 (16.1)	
Others, n (%)	83 (41.5)	26 (57.8)	57 (36.8)	

Migrant patients were significantly younger at the time of transplantation (p = 0.022). The cause of end-stage renal disease was not different (p = 0.063).

Means ± standard deviation are shown; numbers in parentheses indicate percentages.

### Elisa testing

As described in the manufacturer’s manual (Bordier Affinity Products SA, Crissier, Switzerland), human plasma was diluted at 1:200 in provided dilution buffer. The resultant mixture was incubated at the pre-coated plate at 37 °C for 30 minutes. Following a washing procedure using an ELISA washing machine, the conjugate diluted at 1:51 in the provided dilution buffer was incubated for 30 minutes at 37 °C. After a second wash the substrate was reacted for 30 minutes again at 37 °C and finally read by an ELISA reader at 405 nm.

All extinction values were calculated against the reference standard provided with each test package. A test was considered positive when the absorbance of a patient sample divided by the weak positive reference standard was ≥1. Patients classified as uncertain (such as value 0.40 to 0.99) were also monitored. The test sensitivity was measured as 89.5% and specificity as 98.3% in a previous validation study by experienced parasitologists using samples from verified infections^9^.

### Data processing and statistical analyses

We presented categorized data as absolute counts and relative frequencies. Continuous data were presented as means ± standard deviation (SD). Data management and analysis was performed by Microsoft Excel (© Microsoft, Redmont, WA) and GraphPad Prism (© GraphPad Prism version 7.00 for Windows, GraphPad Software, La Jolla California, USA). Differences of *S. stercoralis* IgG signals were analyzed with the non-parametric Mann-Whitney test. Differences concerning the clinical findings, which represent categorical parameters, were compared using Chi-square test. The mean age at transplantation showed a normal distribution and was consequently compared between migrant and non-migrant patients using unpaired sample t-tests. The *S. stercoralis* IgG signals changing over time were analyzed using the paired t-test. All tests performed were two-sided and a P-value of less than 0.05 was considered significant.

### Approval and Registration

The study protocol was performed in accordance to the Helsinki Declaration and approved by the Ethics Committee of the Medical University of Vienna (EK 771/2009). All study participants were adults over 18 years and gave written informed consent. The study was registered at ClinicalTrials.gov (ID: NCT00978965). All methods were performed in accordance with the relevant guidelines and regulations and no organs/tissues were procured from prisoners.

## Results

**Figure 1:**
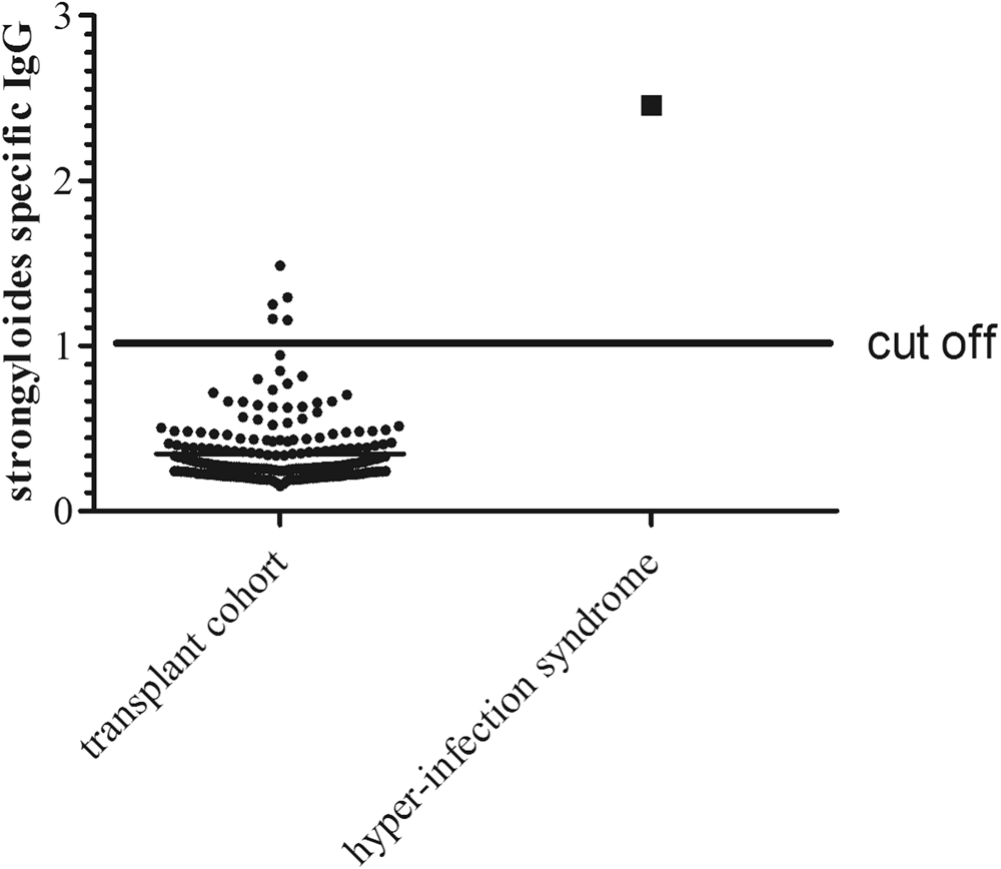
Relative *S. stercoralis* IgG signal among renal allograft recipients (n = 200), one patient with diagnosed hyperinfection syndrome. The results were given as value of absorbance of the individual patient, divided by the value of absorbance obtained from the control reference sample for low infection. Patients above the cut off value of 1 were considered positive.

**Figure 2:**
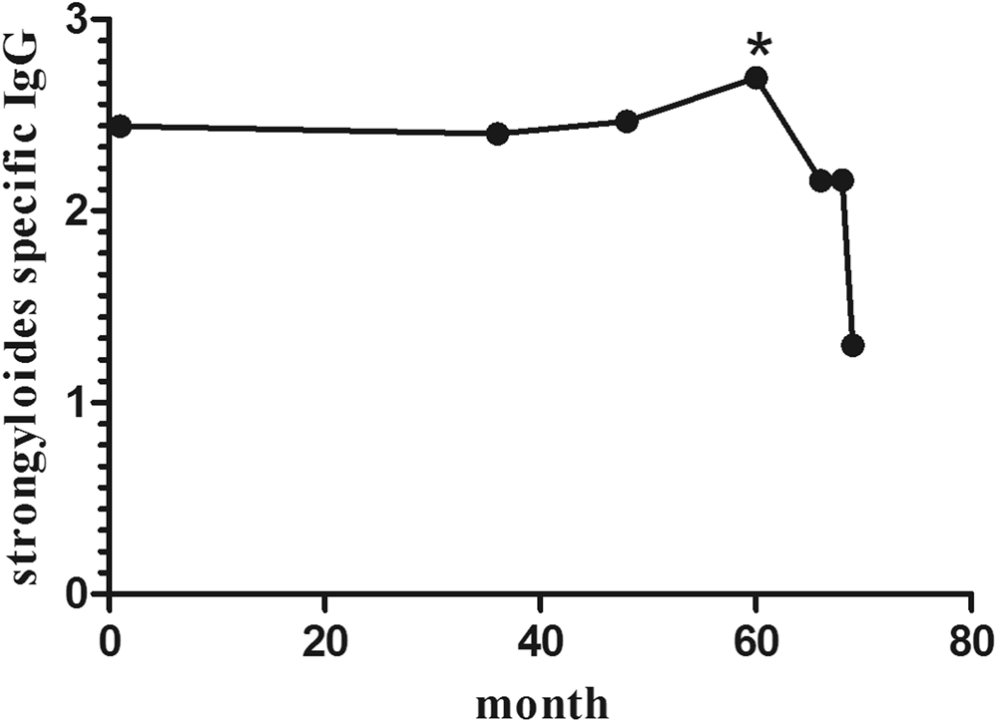
Course of relative *S. stercoralis* IgG signal in one patient with hyperinfection syndrome. The patient developed a hyperinfection syndrome with nearly fatal course in the fifth-year post transplantation following a high-dose steroid treatment. The asterisk (*) indicates the time of diagnosis. The *S. stercoralis* IgG signal gradually decreased after anthelmintic treatment.

Out of 200 renal allograft recipients, six patients (prevalence 3%) were tested positive for *S. stercoralis* infection with a cut off defined as patient extinction value divided by low infection reference value ≥1 (Fig. 1). One out of 6 high signal patients developed a hyperinfection syndrome in the fifth year post transplantation. The patient showed a positive *S. stercoralis* signal already five years before onset of the disease (Fig. 2). None of the other patients testing positive developed a hyperinfection syndrome, although they were kept on the standard triple immunosuppressive regimen. Fifty patients were investigated 12 months after the first testing and the *S. stercoralis* IgG signal significantly decreased (month one: 0.37 ± 0.2 vs. month twelve 0.31 ± 0.13, p = 0.003) (Supplementary Fig. 1).

The hyperinfected patient was diagnosed through histological specimens with eggs and early stages of larvae between intestinal villi in duodenal mucosa by hematoxylin and eosin staining and positive stool testing. Furthermore, larvae were found in the pleural effusion, which were stained by immunofluorescence and visualized by confocal microscopy (see Supplementary Fig. 2). The patient managed to survive the severe illness consisting of weight loss, pneumonitis and sepsis. The recovery time took more than three months. Agar plate testing was used for fecal testing excluding persistence of parasite infection in stool following anthelminthic therapy.

**Figure 3:**
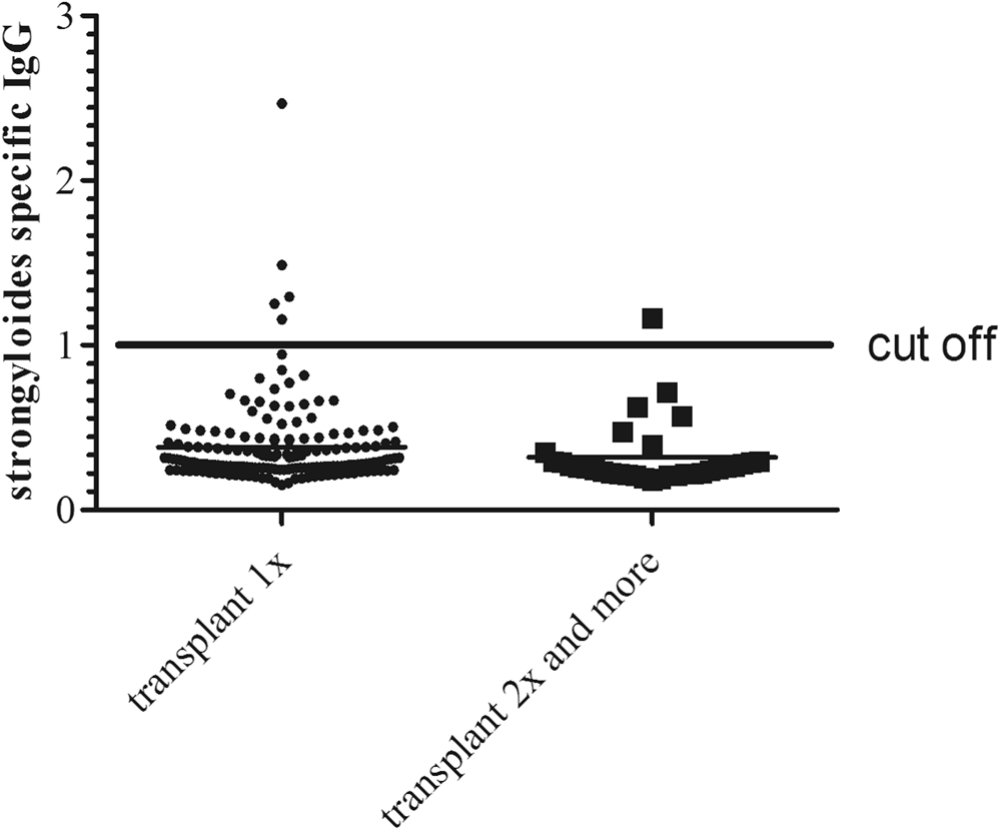
Comparison of the relative *S. stercoralis* IgG signal between first time (n = 167) and repeated renal allograft recipients (n = 33). Patients having received only one transplant had significantly higher *S. stercoralis* IgG signals when compared with repeatedly transplanted patients (0.38 ± 0.02 vs. 0.32 ± 0.04; p = 0.008).

In order to find out whether repetitive allografting combined with immunosuppressive regimens would change *S. stercoralis* IgG signal, we compared the calculated IgG signal of first transplant (0.38 ± 0.02, n = 167) with repeated transplant recipients (0.32 ± 0.04, n = 33). Repeated transplant recipients showed significantly lower signals compared with patients only once transplanted (p = 0.008, Fig. 3).

We further sought whether migrants show a higher prevalence of *S. stercoralis* infection and seropositivity. For this reason, we compared the relative IgG *S. stercoralis* signal of migrants with non-migrants and found higher signals among migrants (0.40 ± 0.05, n = 45) when compared with non-migrants (0.36 ± 0.02, n = 155) but this did not reach statistical significance (p = 0.79, Supplementary Fig. 3).

## Discussion

In this post hoc analysis we find a *Strongyloides stercoralis* seropositivity of 3% among renal allograft transplant patients originating, at least half of them, from Central Europe and the remainder from the former Yugoslavia, Turkey and East Asia and a 16% risk of developing hyperinfection syndrome in seropositive patients. In view of the current literature and supported by our results, screening for *S. stercoralis* infections in both transplant recipients and donors in Austria is justified based on (1) data revealing a not insignificant prevalence of latent infection amongst recipients in nonendemic areas, particularly amongst those of migrant background or travel history (2) there being cases of *Strongyloides* infections transmitted from donors^20,21^ and (3) previous studies demonstrating increased risk of developing hyperinfection syndrome in transplant recipients^22^.

In this study, we found that one patient with hyperinfection syndrome had high IgG signals years before onset of the disease. This does not exclude that he had helminths in his intestine but the antibody titer eventually helped to keep the infection dormant. Only after the start of a high-dose steroid treatment did the systemic spreading appear. Thus, we hypothesize that immunization will not be able to completely prohibit outbreaks of hyperinfection syndromes. In this context, there is a debate about immunization against such parasitic infections. Several immunogenic parasite-specific proteins have already been identified^23–25^ and might be testable for vaccines, but studies in this respect in humans are not yet available.

There is no clear explanation why only one in six patients developed a hyperinfection syndrome and not others who had high IgG *S. stercoralis* signals and received e.g. anti-thymocyte globulin and intermittently high doses of steroids. Differences in infecting parasite virulence is a potential explanation. Another explanation would be that the individual immunogenetic make-up predisposes for a disease outbreak. Recent studies have shown in this context that host genetics is an important factor for the intensity of infection and morbidity due to human helminths^26^. Which of the above factors or possibly a third factor, such as the intensity of immunosuppression, is the main contributor of the outbreak of a hyperinfection syndrome, cannot be answered by our study. Three out of five patients with positive *Strongyloides* serology but without progression into hyperinfection syndrome had a travel history in *Strongyloides* endemic countries (Thailand and Egypt). However, two patients with *Strongyloides* seropositivity had no travel history outside of Austria and were not subsequently affected by clinically confirmed infection or hyperinfection syndrome. Therefore, it cannot be excluded that patients were not infected and test results were false positive.

One of the main problems with an uncommon disease like *S. stercoralis* infection is that symptoms are often nonspecific. The one patient with the hyperinfection syndrome was diagnosed by chance through histopathology obtained by endoscopic biopsy, which had been performed because of weight loss and loss of appetite. In a retrospective analysis, the patient showed a positive *S. stercoralis* signal already five years before the onset of disease with constantly high antibody signals over time. After the delayed histological diagnosis, the patient received three cycles of anthelminthic treatment. While *S. stercoralis* IgG signal was decreasing very slowly, helminths disappeared in stool after therapy. This study demonstrates how clinically nonspecific *Strongyloides* infections can occur. From this point of view, serological testing for *Strongyloides* should be performed more frequently in transplant patients with unexplained symptoms.

Only direct sequencing of *S. stercoralis* might allow verification of which area the parasites were originating from. But thus far only parasites from Asia, Southern America and Africa have been characterized by sequencing^3,4^. Data from Europe are pending.

There are several limitations to the interpretation of the study. There was a significant decrease of relative *S. stercoralis* IgG signal in patients over time post transplantation. A possible explanation could be a reduction in antibody production induced by immunosuppressive drugs. Accordingly, false negative ELISA test results are a known phenomenon in immunocompromised patients^27^. For this reason, a not insignificant false negative rate may occur in high-risk transplant patients (e.g. in patients with travel history who are infected but whose titre has dropped just below the cutoff due to immunosuppression), who remain undetected. Therefore, the actual prevalence of *Strongyloides* infections may be underestimated. This in turn means that anthelminthic treatment could be justified for individuals from endemic countries who have an inconclusive serology. Further studies are required in this field. On the other hand, as similarity is shown at the protein level between different helminths^25^, ELISA test results may also be false-positive due to cross-reactivity with other helminth infections^9^. This is particularly noteworthy in individuals from countries known to be endemic to various helminth parasites. Another limitation is that the assessment of the absolute risk of *Strongyloides* hyperinfection syndrome in patients with latent infection is not likely to be accurate due to given test limitations and the relatively small number of patients with progressive disease. Nevertheless, the testing used is validated and established for diagnosis of *S. stercoralis* infections and prevalence studies^9^. A further important issue is that the anthelmintic effect of ivermectin treatment to prevent *Strongyloides* hyperinfection syndrome in untreated seropositive patients was not the subject of this study, there is however established evidence to support its use^28,29^. Finally, the retrospective design of the study limited the analysis due to predesigned study conditions and therefore availability of selectively preserved samples.

## Conclusion

The novel data from this study show a prevalence of 3% of *S. stercoralis* infection or seropositivity among renal transplant recipients in the non-endemic area of Central Europe, demonstrating that stronglyoidiasis in transplant recipients is an important issue that physicians in Vienna, Austria, should be aware of. Furthermore, our study suggests that recipient and donor screening should be recommended in kidney transplantation programs in Austria and also in other non-endemic countries as infection rates increase due to migration and travel. However, seronegativity does not necessarily exclude *Strongyloides* infection in high-risk immunosuppressed individuals, as a drop in signal is observed in patients with immunosuppression, therefore the prevalence of S*trongyloides* infections may be underestimated. Finally, understanding why some S*trongyloides* seropositive individuals develop hyperinfection syndrome with or without time delay after transplantation and others do not, is currently not well understood and requires further research.

## Electronic supplementary material

Supplementary Figures 1-3

## Data Availability

The datasets generated during and/or analyzed during the current study are available from the corresponding author on request.

## References

[1] Schär Fabian, Trostdorf Ulf, Giardina Federica, Khieu Virak, Muth Sinuon, Marti Hanspeter, Vounatsou Penelope, Odermatt Peter (2013). Strongyloides stercoralis: Global Distribution and Risk Factors. PLoS Neglected Tropical Diseases.

[2] Bisoffi Zeno, Buonfrate Dora, Montresor Antonio, Requena-Méndez Ana, Muñoz Jose, Krolewiecki Alejandro J., Gotuzzo Eduardo, Mena Maria Alejandra, Chiodini Peter L., Anselmi Mariella, Moreira Juan, Albonico Marco (2013). Strongyloides stercoralis: A Plea for Action. PLoS Neglected Tropical Diseases.

[3] Kikuchi T (2016). Genome-Wide Analyses of Individual Strongyloides stercoralis (Nematoda: Rhabditoidea) Provide Insights into Population Structure and Reproductive Life Cycles. PLoS Negl Trop Dis.

[4] Nagayasu E (2017). A possible origin population of pathogenic intestinal nematodes, Strongyloides stercoralis, unveiled by molecular phylogeny. Sci Rep.

[5] Basile A (2010). Disseminated Strongyloides stercoralis: hyperinfection during medical immunosuppression. J Am Acad Dermatol.

[6] Goncalves AL, de Araujo KC, Carvalho EF, Ueta MT, Costa-Cruz JM (2016). Specific IgG and immune complex responses to parthenogenetic females and eggs of nematode Strongyloides venezuelensis for the diagnosis of immunosuppression in infected rats. J Helminthol.

[7] Mejia R, Nutman TB (2012). Screening, prevention, and treatment for hyperinfection syndrome and disseminated infections caused by Strongyloides stercoralis. Curr Opin Infect Dis.

[8] Ramanathan R, Nutman T (2008). Strongyloides stercoralis infection in the immunocompromised host. Curr Infect Dis Rep.

[9] Bisoffi Z (2014). Diagnostic accuracy of five serologic tests for Strongyloides stercoralis infection. PLoS Negl Trop Dis.

[10] Repetto SA (2013). An improved DNA isolation technique for PCR detection of Strongyloides stercoralis in stool samples. Acta Trop.

[11] Taniuchi M (2011). High throughput multiplex PCR and probe-based detection with Luminex beads for seven intestinal parasites. Am J Trop Med Hyg.

[12] Verweij JJ (2009). Molecular diagnosis of Strongyloides stercoralis in faecal samples using real-time PCR. Trans R Soc Trop Med Hyg.

[13] Morgan JS, Schaffner W, Stone WJ (1986). Opportunistic strongyloidiasis in renal transplant recipients. Transplantation.

[14] White JV, Garvey G, Hardy MA (1982). Fatal strongyloidiasis after renal transplantation: a complication of immunosuppression. Am Surg.

[15] Palau LA, Pankey GA (1997). Strongyloides hyperinfection in a renal transplant recipient receiving cyclosporine: possible Strongyloides stercoralis transmission by kidney transplant. Am J Trop Med Hyg.

[16] Nolan TJ, Schad GA (1996). Tacrolimus allows autoinfective development of the parasitic nematode Strongyloides stercoralis. Transplantation.

[17] Belhassen-Garcia M (2017). Surveillance of strongyloidiasis in Spanish in-patients (1998-2014). Plos One.

[18] Hamilton KW (2011). Donor-derived Strongyloides stercoralis infections in renal transplant recipients. Transplantation.

[19] Segarra-Newnham M (2007). Manifestations, diagnosis, and treatment of Strongyloides stercoralis infection. Ann Pharmacother.

[20] Transmission of Strongyloides stercoralis through transplantation of solid organs—Pennsylvania, 2012 (2013). MMWR. Morbidity and mortality weekly report.

[21] Hoy WE (1981). Transmission of strongyloidiasis by kidney transplant? Disseminated strongyloidiasis in both recipients of kidney allografts from a single cadaver donor. Jama.

[22] Martinez-Perez Angela, Roure Díez Silvia, Belhassen-Garcia Moncef, Torrús-Tendero Diego, Perez-Arellano Jose Luis, Cabezas Teresa, Soler Cristina, Díaz-Menéndez Marta, Navarro Miriam, Treviño Begoña, Salvador Fernando (2018). Management of severe strongyloidiasis attended at reference centers in Spain. PLOS Neglected Tropical Diseases.

[23] Kerepesi LA (2005). DNA immunization with Na+-K + ATPase (Sseat-6) induces protective immunity to larval Strongyloides stercoralis in mice. Infect Immun.

[24] Kerepesi LA (2004). Human immunoglobulin G mediates protective immunity and identifies protective antigens against larval Strongyloides stercoralis in mice. J Infect Dis.

[25] Marcilla A (2012). The transcriptome analysis of Strongyloides stercoralis L3i larvae reveals targets for intervention in a neglected disease. PLoS Negl Trop Dis.

[26] Quinnell RJ (2003). Genetics of susceptibility to human helminth infection. Int J Parasitol.

[27] Luvira V (2016). Comparative Diagnosis of Strongyloidiasis in Immunocompromised Patients. Am J Trop Med Hyg.

[28] Hays R, Esterman A, McDermott R (2017). Control of chronic Strongyloides stercoralis infection in an endemic community may be possible by pharmacological means alone: Results of a three-year cohort study. PLoS Negl Trop Dis.

[29] Requena-Mendez A (2017). Evidence-Based Guidelines for Screening and Management of Strongyloidiasis in Non-Endemic Countries. American Journal of Tropical Medicine and Hygiene.

