# Supplementary material for: Prevalence of Strongyloides stercoralis infection and hyperinfection syndrome among renal allograft recipients in Central Europe
Source: Sci Rep. 2018 Oct 18;8:15406. doi: 10.1038/s41598-018-33775-3 (PMC6194009; doi:10.1038/s41598-018-33775-3)
Supplement: Supplementary file 1 — Supplementary Figures 1-3 [file 41598_2018_33775_MOESM1_ESM.docx]

**Prevalence of *Strongyloides stercoralis* infection and hyperinfection syndrome among renal allograft recipients in Central Europe.**

Wolfgang Winnicki^1¶*^, Michael Eder^1¶^, Peter Mazal^2^, Florian J. Mayer^3^, Gürkan Sengölge^1^, Ludwig Wagner^1^

^1^Department of Internal Medicine III; Division of Nephrology and Dialysis, Medical University of Vienna, Vienna, Austria

^2^Department of Clinical Pathology, Medical University of Vienna, Vienna, Austria

^3^Department of Laboratory Medicine, Medical University of Vienna, Vienna, Austria

^*^ Corresponding author

E-mail: wolfgang.winnicki@meduniwien.ac.at

^¶^These authors contributed equally to this work.

**Supplementary Figure 1:**

**Time course of relative *S. stercoralis* IgG signal.** *Strongyloides stercoralis* IgG signals were analyzed at month one and twelve after transplantation (n=50). A decrease of the IgG signal was detected over time (month one: 0.37±0.2, month twelve: 0.31±0.13; p=0.003).


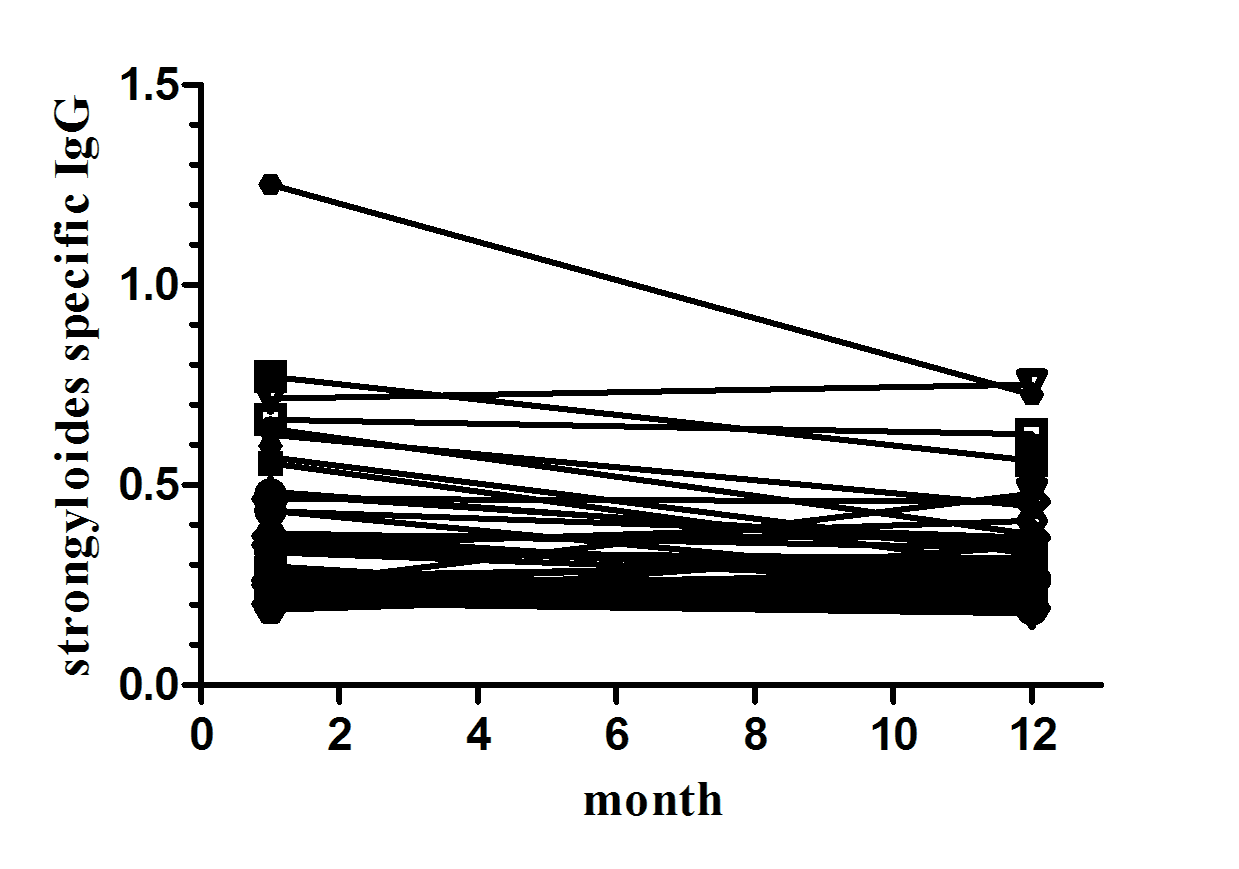


**Supplementary Figure 2:**

***Strongyloides stercoralis* larva in pleural effusion stained by immunofluorescence and visualized by confocal microscopy**. An *S. stercoralis* larva in pleural effusion stained by *S. stercoralis* positive human serum (green) and DNA/nuclei visualization by DAPI (blue).


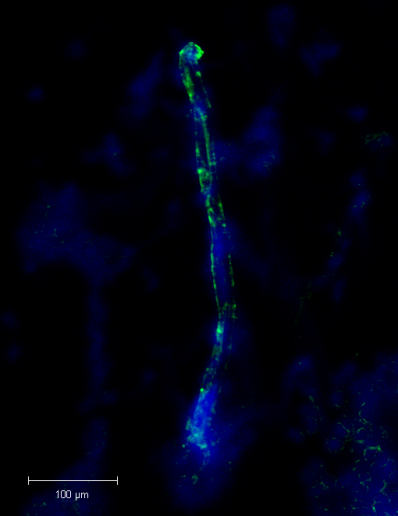


Pleural effusion cytology was prepared using a cytospin preparation from pleural effusion sediment. In brief, 6 mL of pleural effusion was centrifuged at 1500 RPM and the pellet was resuspended in culture medium. One hundred µL of the resultant cell suspension was placed into the funnel of a Shandon Cytocentrifuge (Shandon Southern Instruments, Inc., Sewickley, PA, USA). The cytoslide was air-dried for 2 hours and fixed in acetone for 5 minutes. *S. stercoralis* IgG positive human serum was diluted 1:50 and the cell containing area was overlaid after wetting in PBS with the *S. stercoralis* positive serum, followed by placing a hydrophobic circle around the area with a Dako pen (DAKO, Glostrup, Denmark). In parallel, an *S. stercoralis* negative serum has been used as control. Following an incubation period overnight at 4 °C, slides were washed in PBS and the secondary antibody (rabbit anti-human IgG conjugated with Alexa 488) was incubated for 1 hour at room temperature. 5 minutes before washing, DAPI was added onto the slide for counterstaining DNA/nuclei. After washing in PBS, slides were mounted in Vectashield mounting medium for fluorescence (Vector Laboratories Inc., Burlingame, CA, USA). Images were recorded by confocal microscopy.

**Supplementary Figure 3:**

**Relative *S. stercoralis* IgG signal** **in renal allograft recipients with non-migrant (n=155) and migrant (n=45) background.** There was no statistical significant difference in IgG signals between non-migrants and migrant recipients (0.36 ± 0.02 vs. 0.40 ± 0.05; p=0.79).


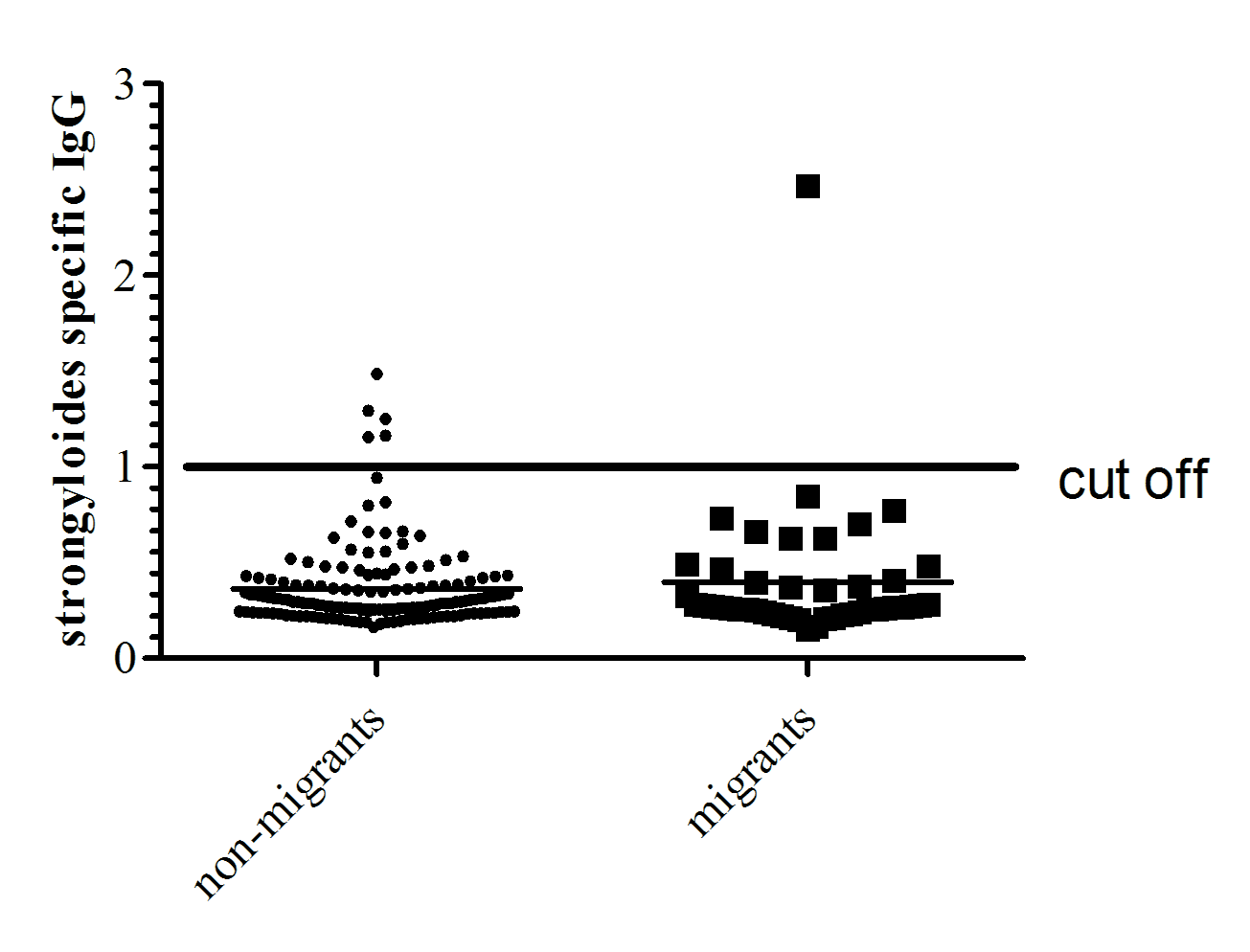


The square with the highest signal in the migrant group represents the patient who has developed a hyperinfection syndrome.
